# The Distinct Transcriptional Response of the Midgut of *Amblyomma sculptum* and *Amblyomma aureolatum* Ticks to *Rickettsia rickettsii* Correlates to Their Differences in Susceptibility to Infection

**DOI:** 10.3389/fcimb.2017.00129

**Published:** 2017-04-28

**Authors:** Larissa A. Martins, Maria F. B. de Melo Galletti, José M. Ribeiro, André Fujita, Francisco B. Costa, Marcelo B. Labruna, Sirlei Daffre, Andréa C. Fogaça

**Affiliations:** ^1^Departamento de Parasitologia, Instituto de Ciências Biomédicas, Universidade de São PauloSão Paulo, Brazil; ^2^Laboratory of Malaria and Vector Research, National Institute of Allergy and Infectious DiseasesRockville, MD, USA; ^3^Departamento de Ciência da Computação, Instituto de Matemática e Estatística, Universidade de São PauloSão Paulo, Brazil; ^4^Departamento de Medicina Veterinária Preventiva e Saúde Animal, Faculdade de Medicina Veterinária e Zootecnia, Universidade de São PauloSão Paulo, Brazil

**Keywords:** spotted fever, tick, rickettsiae, *Rickettsia rickettsii*, *Amblyomma*, midgut, RNA-seq, transcriptome

## Abstract

*Rickettsia rickettsii* is a tick-borne obligate intracellular bacterium that causes Rocky Mountain Spotted Fever (RMSF). In Brazil, two species of ticks in the genus *Amblyomma, A. sculptum* and *A. aureolatum*, are incriminated as vectors of this bacterium. Importantly, these two species present remarkable differences in susceptibility to *R. rickettsii* infection, where *A. aureolatum* is more susceptible than *A. sculptum*. In the current study, *A. aureolatum* and *A. sculptum* ticks were fed on suitable hosts previously inoculated with *R. rickettsii*, mimicking a natural infection. As control, ticks were fed on non-infected animals. Both midgut and salivary glands of all positively infected ticks were colonized by *R. rickettsii*. We did not observe ticks with infection restricted to midgut, suggesting that important factors for controlling rickettsial colonization were produced in this organ. In order to identify such factors, the total RNA extracted from the midgut (MG) was submitted to next generation RNA sequencing (RNA-seq). The majority of the coding sequences (CDSs) of *A. sculptum* differentially expressed by infection were upregulated, whereas most of modulated CDSs of *A. aureolatum* were downregulated. The functional categories that comprise upregulated CDSs of *A. sculptum*, for instance, metabolism, signal transduction, protein modification, extracellular matrix, and immunity also include CDSs of *A. aureolatum* that were downregulated by infection. This is the first study that reports the effects of an experimental infection with the highly virulent *R. rickettsii* on the gene expression of two natural tick vectors. The distinct transcriptional profiles of MG of *A. sculptum* and *A. aureolatum* upon infection stimulus strongly suggest that molecular factors in this organ are responsible for delineating the susceptibility to *R. rickettsii*. Functional studies to determine the role played by proteins encoded by differentially expressed CDSs in the acquisition of *R. rickettsii* are warranted and may be considered as targets for the development of strategies to control the tick-borne pathogens as well as to control the tick vectors.

## Introduction

Rocky Mountain Spotted Fever (RMSF), also known as Brazilian Spotted Fever (BSF), is a severe tick-borne illness caused by *Rickettsia rickettsii*. In Brazil, *R. rickettsii* is transmitted to humans by *Amblyomma sculptum*, formerly named *Amblyomma cajennense* (Nava et al., 2014), and *Amblyomma aureolatum* (Labruna, 2009). The prevalence rates of *R. rickettsii*-infected ticks in endemic areas are low, oscillating around 1%. These low prevalence rates seems to be associated with lower reproductive and survival rates of infected ticks, suggesting that *R. rickettsii* is also pathogenic to its vectors (Burgdorfer, 1988; Niebylski et al., 1999; Labruna et al., 2008). Previous experimental infections with *R. rickettsii* have demonstrated that 80–100% of *A. aureolatum* ticks from a laboratory colony acquire this bacterium, whereas only 10 to 60% of *A. sculptum* ticks become infected (Labruna et al., 2008). These results showed that *A. aureolatum* is more susceptible to rickettsial infection than *A. sculptum*.

The midgut (MG) is the first tick organ that blood meal-acquired microbes (including tick-borne pathogens) interact with. The neutral pH and the nutrients of the host blood favor microbial proliferation in the lumen of the tick MG. In addition, microbes do not directly contact digestive enzymes, as digestion in ticks is intracellular (Sojka et al., 2008). Therefore, to efficiently prevent microbial growth, ticks must present a robust immune response in MG (Hajdusek et al., 2013). Indeed, different antimicrobial peptides (AMPs) and/or their transcripts have been detected in this organ, such as hemocidins (host hemoglobin-derived antimicrobial peptides) (Fogaca et al., 1999; Nakajima et al., 2003; Sonenshine et al., 2005; Belmonte et al., 2012), defensins (Nakajima et al., 2001, 2002; Hynes et al., 2005; Rudenko et al., 2005; Ceraul et al., 2007; Zhou et al., 2007), and lysozymes (Kopacek et al., 1999; Grunclova et al., 2003; Simser et al., 2004; Ceraul et al., 2007). Moreover, transcriptomics data suggest that tick MG contains proteins involved in the production of reactive oxygen species (ROS), which may also help to control microorganism growth (Anderson et al., 2008). To be successfully transmitted to another host, tick-borne pathogens must then be able to resist to the immune reactions of tick MG, transpose this barrier, and reach the salivary glands through hemolymph.

In the current work, we present the first global transcription profile of MG of *A. sculptum* and *A. aureolatum* in response to infection with *R. rickettsii*. Importantly, ticks were infected by feeding on hosts previously inoculated with *R. rickettsii*, mimicking a natural infection. The analyses were performed using next generation RNA sequencing (RNA-seq) and data were validated by quantitative polymerase chain reaction preceded by reverse transcription (RT-qPCR). The proteins encoded by differentially expressed CDSs in the colonization of tick MG by *R. rickettsii* should be functionally characterized and may be considered as targets for the development of strategies to control the tick borne pathogens as well as to control the tick vectors.

## Materials and methods

### Ethics statement

All animal experiments were performed in strict accordance with the Institutional Animal Care and Use Committees from of the Faculty of Veterinary Medicine and Animal Husbandry (protocol number 1423/2008) and the Institute of Biomedical Sciences (protocol number 128/11) of the University of São Paulo, São Paulo, Brazil. Animal purchases and euthanize procedures were performed as described in Galletti et al. (2013).

### *R. rickettsii*-infected and non-infected ticks

Ticks were obtained from laboratory colonies of *A. sculptum* (Pedreira strain; state of São Paulo, Brazil) and *A. aureolatum* (Atibaia strain; state of São Paulo, Brazil). *A. sculptum* larvae, nymphs, and adults were fed on rabbits (*Oryctolagus cuniculus*), as previously described (Pinter et al., 2002). Feeding of *A. aureolatum* developmental stages was accomplished following the methodology detailed in Galletti et al. (2013). To obtain infected ticks, larvae were fed on hosts previously infected with the highly virulent Taiaçu strain of *R. rickettsii*, using the procedure described by Pinter and Labruna (2006) and Galletti et al. (2013). Off-host phases were held in an incubator at 25°C and 95% relative humidity.

Adult ticks from the *R. rickettsii*-infected and non-infected groups were manually removed from the vertebrate hosts after 3 days of feeding. The ticks were washed in 70% ethanol and sterile phosphate-buffered saline (PBS) (10 mM NaH_2_PO_4_, 1.8 mM KH_2_PO_4_, 140 mM NaCl, and 2.7 mM KCl, pH 7.4) for 10 min each. Midgut (MG) and salivary glands (SG) of each tick were dissected and separately transferred to 100 μL of RNA*later*® Solution (ThermoFisher Scientific).

### Nucleic acid extraction

The SG and MG of each adult tick were homogenized and submitted to a simultaneous isolation of genomic DNA (gDNA) and total RNA using the InviTrap® Spin Cell RNA Mini Kit (Stratatec), according to the manufacturer's specifications.

### *R. rickettsii* quantification

gDNA was used to quantify the total number of rickettsiae in tick MG by real-time quantitative PCR (qPCR) using a hydrolysis probe for the citrate synthase gene (*glt*A) of *R. rickettsii*, as previously described by Galletti et al. (2013). All samples were analyzed in three technical replicates. Samples of gDNA extracted from control ticks were also tested to confirm the absence of infection.

### RNA-seq, assembly and annotation

To perform mRNA sequencing (RNA-seq) analysis, total RNA extracted from the MG of adult females were pooled, according to the following description: eight *A. sculptum* harboring 2.2 × 10^7^ ± 2.4 × 10^7^ rickettsiae and ten *A. aureolatum* presenting 4.8 × 10^7^ ± 2.7 × 10^7^ rickettsiae composed the infected samples (AsI and AaI, respectively); and eight non-infected adult females of *A. sculptum* and 10 non-infected *A. aureolatum* composed the control samples (AsC and AaC, respectively). Each tick contributed equally for the composition of the RNA pool samples. Samples were tagged and multiplex sequenced in four lanes using a HiSeq™ sequencing system (Illumina) at the North Carolina State University facility (North Carolina, USA).

Near 567 million reads of 101 base pairs were obtained using the single read mode (these reads also include the transcriptomes of SG of *A. aureolatum* and *A. sculptum*; data not shown). Reads for each species were assembled together using Abyss and Soapdenovo Trans programs with *K*-values varying from 21 to 91 (in 10 interval increments). Resulting assemblies were concatenated and clustered using the blastn tool (performed locally from executables obtained at the NCBI FTP site ftp://ftp.ncbi.nih.gov/blast/executables/) (Altschul et al., 1990) and CAP3 assembler (Huang and Madan, 1999) by a decreasing word size inclusion strategy, as described by Karim et al. (2011), starting at 300 and ending in 60. Coding sequences (CDSs) were extracted as detailed by Karim et al. (2011), based on matches to public databases or longer open reading frames with a signal peptide as an indicative of secretion. Data were organized in a hyperlinked spreadsheet as described by Ribeiro et al. (2004). The blastx (Altschul et al., 1997) tool was used to compare the predict amino acid sequences translated from the nucleotide sequences to the NR protein database of the NCBI and to the Gene Ontology (GO) database (Ashburner et al., 2000). The tool reverse position-specific BLAST (rpsblast) (Altschul et al., 1997) was used to search for conserved protein domains in the Pfam (Bateman et al., 2000), SMART (Schultz et al., 2000), KOG (Tatusov et al., 2003), and conserved domains databases (CDD) (Marchler-Bauer et al., 2002). To identify putative secreted proteins, predicted proteins starting with a methionine residue were submitted to the SignalP server (Nielsen et al., 1997). In addition, the program NetOGlyc (Julenius et al., 2005) was used to predict glycosylation sites on the proteins. The functional annotation of the CDSs was carried out according to the their *e*-values and their order of appearance on the comparisons described above, as detailed in Karim et al. (2011).

To compare the gene expression between samples [*A. sculptum* infected (AsI) vs. non-infected (AsC) and *A. aureolatum* infected (AaI) vs. non-infected (AaC)], paired comparisons of the number of reads hitting each contig were calculated by X^2^ tests to detect significant differences between samples, where the minimum considered value was larger than 5 and *p* < 0.05. Normalized fold-ratios of the sample reads were computed by adjusting the numerator by a factor based on the ratio of the total number of reads in each sample, and adding one to the denominator to avoid division by zero.

The complete dataset of *A. sculptum* and *A. aureolatum* can be downloaded from the National Center for Biotechnology Information (NCBI). The raw data were deposited to the Sequence Read Archives (SRA) of the NCBI under bioprojects numbers PRJNA343654 (*A. sculptum*, raw reads runs SRR4277085, SRR4277086, SRR4277087, SRR4277088, and SRR4277089), and PRJNA344771 (*A. aureolatum*, raw reads runs SRR4301100, SRR4301108, SRR4301110, and SRR4301120). This Transcriptome Shotgun Assembly (TSA) project has been deposited at DDBJ/EMBL/GenBank under the accession GFAA00000000 (for *A. sculptum*) and GFAC00000000 (for *A. aureolatum*). Only CDS representing 90% of known proteins or larger than 250 amino acids were deposited.

When required, phylogenetics and topology analyses of predicted proteins were performed using the program Clustal omega (European Bioinformatics Institute; EMBL-EBI) with default parameters.

### Real-time quantitative PCR preceded by reverse transcription (RT-qPCR)

Five hundred nanogram of the total RNA extracted from the MG of infected (AsI and AaI) or control (AsC and AaC) ticks were treated with RQ1 RNase-free DNase (Promega) and reverse transcribed (RT) in cDNA using M-MLV Reverse Transcriptase (ThermoFisher Scientific), as detailed by manufacturer. Resulting cDNA was used as template in qPCR with the Maxima SYBR Green/ROX qPCR MasterMix (ThermoFisher Scientific) and specific primers for selected CDSs (Supplementary Table 1). Primers were designed using Primer3 (Rozen and Skaletsky, 2000) and synthesized by ThermoFisher Scientific. qPCR was performed on a StepOnePlus™ Real-Time PCR System (ThermoFisher Scientific), using the following program: 95°C for 10 min followed by 40 cycles at 95°C for 15 s, 60°C for 60 s, and 72°C for 20 s. In addition, a melting curve analysis was carried out to check the specificity of the primers. To determine the efficiency of each pair of primers, standard curves were generated using different concentrations of cDNA (400–3.12 ng; 2-fold dilution).

The 2^−ΔΔCt^ equation was utilized to calculate the relative expression of select CDSs in infected vs. non-infected ticks (Livak and Schmittgen, 2001). The CDS of the ribosomal protein S3A was used as reference (Supplementary Table 1). Eight biological replicates from *A. sculptum* and nine from *A. aureolatum* were analyzed. Each biological replicate corresponds to one tick MG. Student's *t*-test was used to statistically validate the differentially expressed CDSs. To verify the reproducibility of both gene expression measurement techniques, we calculated the Pearson's correlation coefficient between RNA-seq and RT-qPCR.

## Results

Initially, adult females of *A. sculptum* and *A. aureolatum* infected with *R. rickettsii* were obtained by a laboratory-controlled experimental infection. The presence of rickettsiae in midgut (MG) and salivary glands (SG) of ticks was assessed by qPCR. Only fifteen among 100 *A. sculptum* adult females (15%) were positively infected, while 27 from 29 *A. aureolatum* adult females (93%) acquired *R. rickettsii*. Importantly, both MG and SG of all infected ticks were colonized by *R. rickettsii*.

The RNA extracted from the MG of control and infected *A. sculptum* and *A. aureolatum* [*A. sculptum* control (AsC), *A. aureolatum* control (AaC), *A. sculptum* infected (AsI), and *A. aureolatum* infected (AaI)] were multiplex sequenced using an Illumina HiSeq platform. The sequencing of *A. scultpum* RNA samples resulted in 324 million reads of 101 base pairs, which were assembled in 9,560 CDSs. For *A. aureolatum*, near 242 million reads were obtained and assembled in 11,906 CDSs (Table 1).

**Table 1:** **Functional classification of CDSs identified in the MG of ***A. sculptum*** and ***A. aureolatum*** by RNA-seq**.

**Functional categories**	***A. sculptum***				***A. aureolatum***			
	**Number of CDSs**	**Number of reads in infected ticks**	**Number of reads in control ticks**	**Sum of mapped reads**	**Number of CDSs**	**Number of reads in infected ticks**	**Number of reads in control ticks**	**Sum of mapped reads**
cytoskeletal	303	6.41E + 05	9.79E + 05	1.62E + 06	326	7.62E + 05	1.49E + 06	2.25E + 06
detoxification	96	1.48E + 05	2.28E + 05	3.76E + 05	228	4.59E + 05	7.41E + 05	1.20E + 06
extracellular matrix	317	9.15E + 05	1.39E + 06	2.30E + 06	341	6.97E + 05	1.48E + 06	2.17E + 06
immunity	138	3.06E + 05	4.72E + 05	7.78E + 05	145	2.52E + 05	4.69E + 05	7.21E + 05
metabolism	551	1.44E + 06	2.56E + 06	4.00E + 06	1,070	2.14E + 06	4.62E + 06	6.76E + 06
nuclear export	32	6.56E + 04	1.05E + 05	1.71E + 05	47	9.11E + 04	1.46E + 05	2.38E + 05
nuclear regulation	208	2.17E + 05	3.55E + 05	5.72E + 05	355	5.28E + 05	7.56E + 05	1.28E + 06
protein export machinery	305	4.35E + 05	6.65E + 05	1.10E + 06	403	6.31E + 05	1.08E + 06	1.71E + 06
protein modification	198	5.13E + 05	9.67E + 05	1.48E + 06	344	6.96E + 05	1.41E + 06	2.11E + 06
proteasome machinery	178	2.76E + 05	4.61E + 05	7.38E + 05	269	5.68E + 05	8.90E + 05	1.46E + 06
protein synthesis	288	2.11E + 06	3.90E + 06	6.01E + 06	438	2.44E + 06	4.36E + 06	6.80E + 06
secreted	2,535	4.13E + 06	6.11E + 06	1.02E + 07	2,523	4.83E + 06	7.43E + 06	1.23E + 07
signal transduction	825	1.02E + 06	1.38E + 06	2.39E + 06	1,051	1.23E + 06	2.19E + 06	3.42E + 06
storage	20	4.74E + 05	6.72E + 05	1.15E + 06	13	1.89E + 05	2.86E + 05	4.75E + 05
transposon element	576	1.34E + 05	1.52E + 05	2.86E + 05	518	2.02E + 05	3.86E + 05	5.87E + 05
transcription factor	133	1.20E + 05	1.60E + 05	2.81E + 05	172	1.55E + 05	2.79E + 05	4.34E + 05
transcription machinery	430	8.73E + 05	1.37E + 06	2.25E + 06	680	2.00E + 06	2.00E + 06	4.00E + 06
transporter and channels	420	4.49E + 05	5.81E + 05	1.03E + 06	473	5.22E + 05	1.01E + 06	1.53E + 06
unknown conserved	1,035	1.31E + 06	1.89E + 06	3.20E + 06	1,568	2.18E + 06	2.69E + 06	4.87E + 06
unknown	933	9.05E + 05	1.37E + 06	2.28E + 06	907	1.10E + 06	9.77E + 05	2.08E + 06
virus	39	4.56E + 04	4.59E + 04	9.15E + 04	35	3.59E + 04	7.19E + 04	1.08E + 05
TOTAL	9,560	1.65E + 07	2.58E + 07	4.23E + 07	11,906	2.17E + 07	3.48E + 07	5.65E + 07

To identify coding sequences (CDSs) differentially expressed by infection with *R. rickettsii*, we compared the number of reads mapped to each contig in infected vs. non-infected *A. sculptum* (Table 2 and Supplementary Tables 2, 3) and *A. aureolatum* (Table 3 and Supplementary Tables 4, 5). Infection modulated 479 CDSs in the MG of *A. sculptum*, among which 416 were upregulated (Supplementary Table 3) and only 63 were downregulated (Supplementary Table 2). Differently, from 310 CDSs of *A. aureolatum* modulated by infection, 237 were downregulated (Supplementary Table 4) and only 73 were upregulated (Supplementary Table 5). In order to validate the transcription pattern obtained by RNA-seq, selected CDSs were analyzed by RT-qPCR (Tables 2, 3 for *A. sculptum* and *A. aureolatum*, respectively). The correlation between transcriptional data obtained by these two methods was 0.69 (*p* = 0.037) for *A. sculptum* and 0.73 (*p* = 0.039) for *A. aureolatum*, strengthening the transcriptional data obtained by RNA-seq.

**Table 2:** **Selected CDSs of ***A. sculptum*** midgut differentially expressed by infection with ***R. rickettsii*****.

**CDS**	**Annotation**	**RNA-seq**	**RT-qPCR**
		**Fold-change**	**Fold-change**
Acaj-52926	superoxide dismutase	0.17	NA
Acaj-76542	structural constituent of cuticle	0.03	0.19
Acaj-75348	cuticle protein	0.03	NA
Acaj-80620	cuticle protein	0.02	NA
Acaj-81446	chitin binding peritrophin	0.13	NA
AcajSigP-85359	peritrophin	0.17	215.94^**^
Acaj-36823	tumor necrosis factor receptor-associated factor	0.14	NA
Acaj-43466	tumor necrosis factor receptor-associated factor	0.05	NA
Acaj-70988	tumor necrosis factor receptor-associated factor	0.16	NA
Acaj-51987	tumor necrosis factor receptor-associated factor	6.06	NA
Acaj-33728	lysozyme	163.20	NA
Acaj-48379	tick defensin	5.31	4.90^*^
Acaj-74395	antimicrobial peptide	11.24	47.50^**^
Acaj-60304	peptidoglycan recognition protein	8.85	18.31^*^
AcajSigP-81204	peptidoglycan recognition protein	5.83	NA
AcajSigP-6765	ML domain protein	11.68	85.90^**^
AcajSigP-19478	vitellogenin receptor	0.13	NA
AcajSigP-26714	vitellogenin receptor	0.13	0.11^*^
AcajSigP-74567	glycolate oxidase	9.13	NA
AcajSigP-79349	glycolate oxidase	8.05	38.05^*^
Acaj-72627	serine carboxypeptidase	31.63	NA
Acaj-72628	serine carboxypeptidase	33.88	NA
Acaj-72629	serine carboxypeptidase	47.20	NA
Acaj-25905	BPTI/Kunitz domain-containing protein	9.15	NA
Acaj-32963	tick_Kunitz_108	13.74	NA
Acaj-62359	tick_Kunitz_108	6.96	NA
Acaj-67782	Kunitz-type peptidase inhibitor	271.74	NA
Acaj-84823	tick_Kunitz_101	6.04	NA
AcajSigP-26186	tick_Kunitz_115	8.85	NA
AcajSigP-45672	tick_Kunitz_101	8.27	NA
AcajSigP-57222	Kunitz domain protein	7.99	NA
Acaj-39037	secreted mucin MUC17	0.15	NA
AcajTE-85265	hypothetical protein PGCG_00421	0.03	0.10
AcajSigP-78265	hypothetical secreted protein	13.22	3.42

Only CDSs with statistically significant differences in expression in the midgut of infected vs. control (non-infected) ticks, identified by RNA-seq analysis, are presented. Some CDSs were additionally analyzed by RT-qPCR (*p < 0.05 and **p < 0.01; Student's t-test). NA: relative expression not analyzed by RT-qPCR.

**Table 3:** **Selected CDSs of ***A. aureolatum*** midgut differentially expressed by infection with ***R. rickettsii*****.

**CDS**	**Annotation**	**RNA-seq**	**RT-qPCR**
		**Fold-change**	**Fold-change**
Ambaur-58882	cuticular protein	0.08	NA
Ambaur-64626	cuticle protein	0.18	NA
Ambaur-18924	peptidoglycan recognition protein	5.08	NA
Ambaur-45342	lysozyme	5.15	4.06^**^
AmbarSigP-48279	ixoderin precursor	5.29	NA
Ambaur-58673	tumor necrosis factor receptor-associated factor	0.19	NA
AmbarSigP-52021	glucose dehydrogenase	0.04	NA
AmbarSigP-66386	glucose dehydrogenase	0.11	NA
Ambaur-5106	glutamine synthetase	0.15	0.33
Ambaur-61820	homocysteine S-methyltransferase	25.55	1.40
Ambaur-53938	chymotrypsin-elastase inhibitor ixodidin	0.16	NA
Ambaur-58705	serine carboxypeptidase	0.08	NA
Ambaur-60941	secreted mucin MUC17	0.19	NA
Ambaur-63341	secreted mucin MUC17	0.08	NA
Ambaur-63342	secreted mucin MUC17	0.12	NA
AmbarSigP-64116	secreted mucin	0.18	NA
Ambaur-19862	5.3 kda antibacterial peptide	0.09	NA
AmbarSigP-15638	serine carboxypeptidase	0.05	NA
AmbarSigP-13149	serine carboxypeptidase	33.46	NA
AmbarSigP-19966	Kunitz/Bovine pancreatic trypsin inhibitor domain	0.03	NA
AmbarSigP-54335	BPTI/Kunitz domain-containing protein 5	0.03	NA
Ambaur-28274	Kunitz-type protease inhibitor	0.01	NA
Ambaur-43435	BPTI/Kunitz family of serine protease inhibitors	0.02	NA
Ambaur-59675	BPTI/Kunitz domain-containing protein 5	5.09	NA
Ambaur-54534	secreted metalloprotease	0.17	1.00
Ambaur-25926	evasin	11.21	1.39
AmbarSigP-64894	tick transposon	0.18	0.34^*^
AmbarSigP-70666	ceramidase	0.12	0.33^*^
Ambaur-62064	hypothetical protein PGCG_00421	56.03	1.52
Ambaur-4532	ABC transporter	14.06	1.76

Only CDSs with statistically significant differences in expression in the midgut of infected vs. control (non-infected) ticks, identified by RNA-seq analysis, are presented. Some CDSs were additionally analyzed by RT-qPCR (*p < 0.05 and **p < 0.01; Student's t-test). NA: relative expression not analyzed by RT-qPCR.

**Figure 1:**
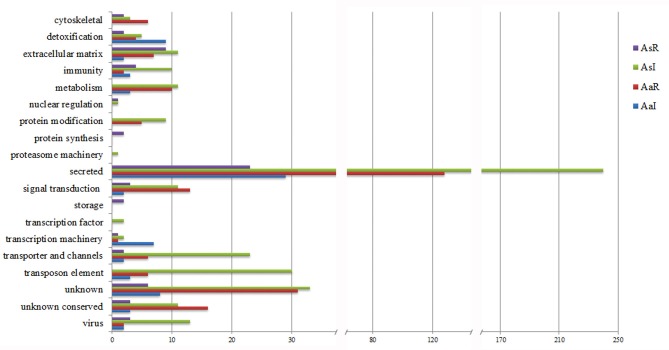
**Functional classification of CDSs differentially expressed in the midgut of ***R. rickettsii***-infected ticks**. AsR, downregulated CDSs of *A. sculptum*; AsI, upregulated CDSs of *A. sculptum*; AaR, downregulated CDSs of *A. aureolatum*; AaI, upregulated CDSs of *A. aureolatum*.

Most of the CDSs upregulated by *R. rickettsii* infection in *A. sculptum* belongs to metabolism, protein modification, secreted, signal transduction, transporter and channels, and transposon elements functional categories (Figure 1). Differently, CDSs in these same functional categories were majorly downregulated in *A. aureolatum*. Moreover, CDSs of *A. sculptum* in protein synthesis and storage categories were majorly downregulated by infection, while CDSs in immunity, proteasome, transcription factor, and virus categories were mostly upregulated. In *A. aureolatum*, CDSs in cytoskeletal category were remarkably downregulated by infection, whereas CDSs in transcription machinery and detoxification categories were upregulated (Figure 1).

In metabolism category, 11 CDSs were upregulated by infection in *A. sculptum* (Supplementary Table 3), while none was downregulated (Supplementary Table 2). On the other hand, 10 CDSs inside this same category were downregulated in *A. aureolatum* (Supplementary Table 4), while three were upregulated (Supplementary Table 5). Among them, only one share the same annotation (pancreatic lipase-like) in both *A. sculptum* (Acaj-69206) and *A. aureolatum* (Ambaur-63833). In addition, two sequences coding glucose dehydrogenases were downregulated in *A. aureolatum* (AmbarSigP-52021 and AmbarSigP-66386; Table 3 and Supplementary Table 4). In *A. sculptum*, two glycolate oxidase CDSs (AcajSigP-74567 and AcajSigP-79349) were upregulated (Table 2 and Supplementary Table 3).

In protein modification category, three CDSs of serine carboxipeptidases (Acaj-72629, Acaj-72628, and Acaj-72627) were upregulated by infection in *A. sculptum* MG (Table 2 and Supplementary Table 3), while none was downregulated. Differently, in *A. aureolatum*, two serine carboxipeptidases CDSs (Ambaur-58705 and AmbarSigP-15638, this last one included in secreted category) were downregulated (Table 3 and Supplementary Table 4) and one (AmbarSigP-13149) was upregulated (Table 3 and Supplementary Table 5). Moreover, 15 metalloprotease CDSs (predicted to be secreted or not) were upregulated in *A. sculptum* (Supplementary Table 3), while eight were downregulated in *A. aureolatum* (Supplementary Table 4). None metalloprotease CDS was detected to be downregulated in *A. sculptum* or upregulated in *A. aureolatum*. Phylogenetics and amino acid alignment analysis showed that, among the metalloproteinases of *A. aureolatum*, six are reprolysins and two are astacins (Supplementary Figure 1). In relation to the metalloproteases of *A. sculptum*, 14 are reprolysins and only one is astacin (Supplementary Figure 1).

Among the differentially expressed sequences that code secreted proteins with annotated function, we highlight lipocalins, serine proteinase inhibitors, principally members of Kunitz family, and mucins. Twenty six lipocalin CDSs were upregulated in *A. sculptum* MG (Supplementary Table 3) and only two in *A. aureolatum* (Supplementary Table 5). On the other hand, 30 lipocalin CDSs were downregulated in *A. aureolatum* (Supplementary Table 4), while none was downregulated in *A. sculptum*. In addition, five sequences encoding Kunitz inhibitors were downregulated by infection in *A. aureolatum* (Table 3 and Supplementary Table 4) and only one was upregulated (Table 3 and Supplementary Table 5). An opposite panorama was observed in *A. sculptum*, with eight CDSs of Kunitz inhibitors upregulated by *R. rickettsii* infection (Table 2 and Supplementary Table 3) and none downregulated. In relation to sequences encoding mucins, one CDS (Acaj-39037) was detected to be downregulated in *A. sculptum* by RNA-seq analysis (Table 2 and Supplementary Table 2). In *A. aureolatum*, four secreted mucin CDSs (Ambaur-60941, Ambaur-63342, Ambaur-63341, and AmbarSigP-64116) were downregulated (Table 3 and Supplementary Table 4).

In signal transduction category, *R. rickettsii* upregulated the transcription of 11 CDSs in *A. sculptum* (Supplementary Table 3) and downregulated 13 CDSs in *A. aureolatum* (Supplementary Table 4). Two sequences inside this category, coding K^+^-dependent Ca^2+^/Na^+^ exchanger NCKX1, are common to both *A. sculptum* (AcajSigP-12807) and *A. aureolatum* (Ambaur-41603). Transporter and channels category also contains CDSs of *A. sculptum* that were upregulated by infection as well as CDSs of *A. aureolatum* that were downregulated (Supplementary Tables 3, 4, respectively). Nonetheless, none of those CDSs shares the same annotation in both tick species.

Cuticle proteins CDSs, which belong to external matrix category, were downregulated by infection in both *A. sculptum* (Acaj-76542, Acaj-80620, and Acaj-75348; Table 2 and Supplementary Table 2) and *A. aureolatum* (Ambaur-64626 and Ambaur-58882; Table 3 and Supplementary Table 4). In this same category, two peritrophin CDSs (Acaj-81446 and Acaj-SigP85359) were downregulated exclusively in infected *A. sculptum* (Table 2 and Supplementary Table 2).

In *A. sculptum*, infection downregulated two vitellogenin receptor CDSs (AcajSigP-26714 and AcajSigP-19478), which are comprised in storage category (Table 2 and Supplementary Table 2). Moreover, two CDSs in transcription factors (Acaj-72151: alcohol dehydrogenase transcription factor Myb/SANT-like; and Acaj-62642: transcription factor NERF) and one in proteasome machinery (AcajTE-85582: E3 ubiquitin -protein ligase RBBP6-like isoform X3) were exclusively upregulated in *A. sculptum* (Supplementary Table 3). In *A. aureolatum*, nine CDSs in detoxification category were upregulated by infection, among which seven code cytochrome P450 (Supplementary Table 5). In *A. sculptum*, two cytochrome P450 CDSs (AcajSigP-19690 and AcajSigP-26888) were also upregulated by infection (Supplementary Table 3), while one superoxide dismutase (SOD) CDS (Acaj-52926) was downregulated (Supplementary Table 2). Seven CDSs in transcription machinery category, among which six code the regulator of rDNA transcription protein 15, were also upregulated by *R. rickettsii* in *A. aureolatum* MG (Supplementary Table 5).

Infection upregulated a higher number of CDS of immune-related proteins in *A. sculptum* than in *A. aureolatum* (Figure 1). Lysozyme, ixoderin, and peptidoglycan recognition protein (PGRP) CDSs were upregulated by infection in both tick species (Tables 2, 3, and Supplementary Tables 3, 5). In *A. sculptum, R. rickettsii* also upregulated the expression of two CDSs (Acaj-48379 and Acaj-74395) that code antimicrobial peptides (AMPs) similar to the defensin of *Amblyomma americanum*, named amercin (Table 2 and Supplementary Table 3). In addition, one CDS of a protein containing ML domain (MD-2-related lipid-recognition) (AcajSigP-6765) was upregulated in *A. sculptum* by infection (Table 2 and Supplementary Table S3). In *A. aureolatum*, sequences coding one 5.3 kDa AMP (Ambaur-19862) and one AMP similar to the ixodidin of *Rhipicephalus microplus* (Ambaur-53938), which exhibit chymotrypsin-elastase inhibitory activity, were downregulated (Table 3 and Supplementary Table 4). In *A. sculptum*, three tumor necrosis factor receptor-associated factor (TRAF) CDSs (Acaj-43466, Acaj-36823, and Acaj-70988; Table 2 and Supplementary Table 2) were downregulated, while one was upregulated (Acaj-51987; Table 2 and Supplementary Table 3). In *A. aureolatum*, only one CDS encoding TRAF (Ambaur-58673) was detected as downregulated (Table 3 and Supplementary Table 4).

As expected, several differentially expressed sequences encode hypothetical proteins or unknown products in both tick species (Figure 1 and Supplementary Tables 2–5). Remarkably, the majority of those sequences encode proteins that are predicted to be secreted in *A. sculptum* (140 among 204 sequences) and in *A. aureolatum* (61 among 117 sequences).

## Discussion

In the current study, laboratory colonies of two different species of ticks that are vectors of *R. rickettsii* in Brazil were fed on infected hosts, mimicking a natural infection. While 93% of *A. aureolatum* females acquired *R. rickettsii*, only 15% of *A. sculptum* females were positively infected. This result reinforces the differences in susceptibility of these two tick species to infection with *R. rickettsii*, as previously reported by Labruna et al. (2008).

It is known that pathogens acquired within the blood meal have to overcome several barriers in ticks to be successfully transmitted to another host (Kopacek et al., 2010; Hajdusek et al., 2013). Firstly, the pathogen must colonize MG and later the salivary glands (SG) through hemolymph. Our data showed that both MG and SG of all positively infected *A. aureolatum* and *A. sculptum* ticks were colonized by *R. rickettsii*. We did not observed ticks with infection restricted to MG, suggesting that if this barrier is broken, this pathogen is able to reach the SG. Therefore, the tick MG probably plays a key role in controlling rickettsial infection. To get better insights on the factors that control infection in tick MG, the global gene expression profile of infected vs. non-infected (control) ticks was determined by RNA sequencing (RNA-seq) and validated using RT-qPCR. The correlation between RNA-seq and RT-qPCR (around 70%) strengthened the transcriptional findings of the current study.

Most CDSs of *A. aureolatum* that were differentially expressed by infection with *R. rickettsii* were downregulated (87%). On the contrary, the majority of CDSs of infected *A. sculptum* were induced (76%). Interestingly, many upregulated CDSs of *A. sculptum* belong to the same functional categories of most downregulated CDSs of *A. aureolatum*, for instance, metabolism, protein modification, and secreted proteins. Further studies are required to determine the role played by CDSs within those functional categories in delineating the differences in susceptibility of these two tick species to infection.

Among CDSs associated to metabolism that were induced by infection in *A. sculptum*, we highlight two glycolate oxidases. In plants, this enzyme converts glycolate to oxalate producing hydrogen peroxide (H_2_O_2_) (Rojas and Mysore, 2012). The upregulation of such oxidases upon infection might represent an attempt of ticks to control *R. rickettsii* through production of H_2_O_2_. Indeed, production of ROS, including superoxide (${\text{O}}_{2}^{-}$) and H_2_O_2_, is an ancient immune response against invader pathogens (Zug and Hammerstein, 2015). It was previously reported that *R. rickettsii* infection induces a pro-oxidant response in human endothelial cells (Santucci et al., 1992; Devamanoharan et al., 1994), causing oxidative cell injury (Eremeeva et al., 2001). The stimulation of hemocytes of the tick *R. microplus* with the Gram-positive bacterium *Micrococcus luteus* also leaded to production of ROS. This activity was abolished by addition of superoxide dismutase (SOD) and catalase, suggesting that both superoxide and H_2_O_2_ are produced upon microbial challenge (Pereira et al., 2001). Besides upregulation of glycolate oxidase, one SOD CDS was downregulated by infection in *A. sculptum*, strengthening that the MG is under oxidative stress upon infection. In *A. aureolatum*, two glucose dehydrogenase encoding genes were downregulated. This enzyme reduces NADP to NADPH. Electrons from NADPH can be used by glutathione reductase to regenerate the intracellular pool of reduced glutathione (Mishra and Imlay, 2012), which, in turn, is important to reduce H_2_O_2_ into H_2_O by action of glutathione peroxidase (Wang et al., 2013). Alternatively, intracellular NADPH can act as the electron donor to the NADPH oxidase complex to catalyze the reduction of molecular oxygen to superoxide in phagocyte membranes (Wang et al., 2013). Therefore, glucose dehydrogenase play an important role in maintaining the cellular redox homeostasis. In a previous study, our research group showed that antioxidant enzymes of *R. rickettsii* (thioredoxin peroxidase 1, glutaredoxin 3, ferredoxin, and also one hypothetical protein A1G_00185 with a thioredoxin domain) are upregulated in MG of fed *A. aureolatum*, which may protect *R. rickettsii* against the deleterious effects of free radicals (Galletti et al., 2013). Then, it is plausible to suppose that the MG of fed infected *A. aureolatum* is under oxidative stress.

It is known that the main proteolytic activities in the MG of ticks are acidic aspartic and cysteine proteinases, whereas exopeptidases (the cysteine amino- and carboxy-dipeptidases cathepsins C and B, respectively) may participate in a later stage of digestion (Horn et al., 2009; Cruz et al., 2010). In addition, two monopeptidases, namely serine carboxypeptidase (Motobu et al., 2007) and leucine aminopeptidase (Hatta et al., 2006), would be possibly involved in the final stage of digestion, liberating free amino acids (Horn et al., 2009). Digestion of hemoglobin, as well as other proteins within the blood meal, is important not only to provide nutrients and energy for molting and vitellogenesis processes, but also to generate fragments with antimicrobial properties (Fogaca et al., 1999; Nakajima et al., 2003; Sonenshine et al., 2005; Belmonte et al., 2012). These antimicrobial fragments, named hemocidins, are probably involved in protection of tick MG against proliferation of pathogens (Kopacek et al., 2010; Hajdusek et al., 2013). We did not detect the transcriptional regulation of aspartic and cysteine proteinases by infection. However, three serine carboxipeptidase CDSs were upregulated in *A. sculptum*, while two were downregulated in *A. aureolatum*. As serine carboxypeptidases are involved in the final stage of hemoglobin digestion (Motobu et al., 2007), studies to determine the contents of hemocidins in MG of *Amblyomma* ticks are warranted to determine the role of these peptides in controlling infection.

Infection also modulated the expression of metalloproteases in tick MG, upregulating 15 CDSs in *A. sculptum* and downregulating eight CDSs in *A. aureolatum*. No metalloprotease CDS was detected to be upregulated in *A. aureolatum* or downregulated in *A. sculptum*. It is known that metalloproteinases of tick saliva, which belong to metzincin family, are essential for both the initial and late feeding stages (Francischetti et al., 2003, 2005a). During the initial feeding stage, these enzymes inhibit blood clotting and degrade extracellular matrix proteins, which is essential for the preparation of the feeding site. As metalloproteases also present anti-angiogenic activity, they are also important in the late feeding stage, inhibiting host tissue repair. Metalloproteases from metzincin family present a zinc-binding consensus motif (HEXXHXXG/NXXH/D, where X corresponds to any amino acid residue) in the active site and a conserved methionine-containing turn (Met-turn) that underlies the active site. According to their structure, metzincins are subdivided in other families, among which are reprolysin and astacin family (Gomis-Ruth, 2009), where tick metalloproteases are included. It was previously reported that reprolysins are majorly expressed in the SG of the tick *R. microplus*, while astacin transcripts are more abundant in the MG and ovaries (Barnard et al., 2012). Importantly, the knockdown of both reprolysins and astacins leaded to a reduction of both oviposition and egg weight, demonstrating that those proteins correspond to promising targets for development of strategies to protect the cattle against ticks (Barnard et al., 2012). Indeed, the immunization of bovines with the reprolysin BrRm-MP4 diminished the feeding and reproductive rates of *R. microplus* females (Ali et al., 2015), strengthening the potential of metalloproteinases as vaccine candidates. Among the metalloproteinases from *A. aureolatum*, six are reprolysins and two are astacins, while 14 of the metalloproteases of *A. sculptum* are reprolysins and only one is astacin.

Many CDSs of both annotated and hypothetical/unknown function proteins that contain signal peptide for secretion were modulated by infection. Importantly, most of the proteins predicted to be secreted were downregulated in *A. aureolatum* and induced in *A. sculptum*. Among them, lipocalins, proteins containing Kunitz domain, and mucins are prominent. *R. rickettsii* infection upregulated 26 lipocalin CDSs in *A. sculptum* and downregulated 30 CDSs in *A. aureolatum*. It is known that lipocalins bind histamine and serotonin (Paesen et al., 1999; Sangamnatdej et al., 2002; Francischetti et al., 2009), which in high concentrations on the feeding pool can affect attachment, feeding efficiency, and reproductive success (Kemp and Bourne, 1980; Paine et al., 1983). Therefore, these anti-inflammatory proteins are extensively detected to be present in tick saliva (Francischetti et al., 2005b; Ribeiro et al., 2006; Alarcon-Chaidez et al., 2007; Karim et al., 2011; Karim and Ribeiro, 2015). The unique non-salivary gland lipocalin described in ticks is savicalin from hemocytes of *Ornithodoros savignyi*. Importantly, savicalin transcripts were also detected to be present in MG and ovaries (Cheng et al., 2010). Therefore, additional studies should be addressed to determine the site of production and the role played by lipocalins in protection of *Amblyomma* ticks against infection.

In addition, CDSs of secreted proteins encoding serine protease inhibitors, predominantly Kunitz inhibitors, were upregulated in *A. sculptum* and downregulated in *A. aureolatum*. Serine proteinase inhibitors were already enrolled in immune reactions of arthropods, mediating both coagulation and melanization processes of the hemolymph and also the production of AMPs (Gulley et al., 2013). These inhibitors may also control pathogen proliferation by inhibiting proteinases they use to colonize the host tissues and evade immune system (Armstrong, 2001). In addition, Kunitz inhibitors are widely described to be produced by ticks, inhibiting blood coagulation and facilitating tick feeding (Corral-Rodriguez et al., 2009). The differential expression of tick Kunitz inhibitors by *Dermacentor variabilis* upon an infection with the avirulent *Rickettsia montanensis* was previously reported (Ceraul et al., 2008). Moreover, it was shown that this Kunitz inhibitor exhibits a bacteriostatic effect against *R. montanensis* (Ceraul et al., 2008) and that its transcription knockdown leads to an increase in the susceptibility of *D. variabilis* to infection (Ceraul et al., 2011).

Some mucins predicted to be secreted were also detected to be differentially expressed in infected ticks. These proteins are lipid binding proteins that constitute an acellular barrier that promote protection of the MG epithelium. Mucins were already reported to be present in MG of ticks, such as *D. variabilis* (Anderson et al., 2008), as well as in SG (Karim et al., 2011; Ribeiro et al., 2011). Interestingly, *D. variabilis* mucins exhibit similarity to insect peritrophins (Anderson et al., 2008). Two peritrophin CDSs and one secreted mucin CDS were detected to be downregulated in *A. sculptum* by RNA-seq analysis. Peritrophin encoding sequences were not detected to be modulated by infection in *A. aureolatum*. However, four secreted mucin CDSs were downregulated in this tick species. It was previously reported that the knockdown of the peritrophin 1 of *Ixodes scapularis* reduces the thickness of the peritrophic matrix, impairing the colonization of the tick MG by *Borrelia burgdorferi*, causative agent of Lyme disease (Narasimhan et al., 2014). Similar effects of peritrophin 1 knockdown was observed when ticks were treated with antibiotics, altering the MG microbiota (Narasimhan et al., 2014). Indeed, ticks harbor a microbiota that can be modified, for instance, upon the treatment with antibiotics (Narasimhan et al., 2014; Clayton et al., 2015; Abraham et al., 2017). Interestingly, a recent study showed that infection with *Anaplasma phagocytophilum*, causative agent of human granulocytic anaplasmosis, also alter the composition of the microbiota of *I. scapularis* MG through the upregulation of the tick anti-freeze glycoprotein (iafgp) (Abraham et al., 2017). Iafgp binds bacterial peptidoglycan, negatively affecting biofilm formation and, consequently, altering microbiota. Differentially from the effect previously observed for *B. burgdorferi* (Narasimhan et al., 2014), the remodeling of the microbiota of *I. scapularis* MG, which diminishes the thickness peritrophic matrix, increased the load of *A. phagocytophilum* (Abraham et al., 2017). It is possible that the MG of *A. sculptum* and *A. aureolatum* harbor a distinct microbiota, which might play a role in delineating susceptibility to infection. In addition, the distinct transcriptional profile triggered by *R. rickettsii* infection may also exert a different impact on the MG microbiota of these two ticks, resulting in differences in susceptibility. Then, studies to determine the composition of the microbiota of the MG of *A. sculptum* and *A. aureolatum* infected or not with *R. rickettsii* are warranted.

It is known that *R. rickettsii* exerts a detrimental effect on ticks, reducing both survival and reproductive rates (Burgdorfer and Brinton, 1975; Niebylski et al., 1999; Labruna et al., 2008; Soares et al., 2012). Our data showed that CDSs of cuticle proteins were downregulated by infection in both *A. sculptum* and in *A. aureolatum*. The expansion of the cuticle is crucial for the engorgement of the tick female (Flynn and Kaufman, 2011), assuring that an enough amount of blood be acquired for egg production. In addition to cuticle proteins, two vitellogenin receptor CDSs were detected to be downregulated by infection in *A. sculptum*. Therefore, it is possible that the downregulation of cuticle proteins and vitellogenin receptors might be involved in the reduction of both oviposition and survival rates in infected ticks.

Infection modulated CDSs of immune-related proteins in both species of ticks. Ixoderins, which are recognition proteins that possesses a C-terminal domain with a high homology to fibrinogen and fibrinogen-related proteins (FREPs), were upregulated by infection in both *A. sculptum* and *A. aureolatum*. Two isoforms of ixoderins, A and B, were identified in the hard tick *Ixodes ricinus* (Rego et al., 2005) and possess similarity to the lectin Dorin M of the soft tick *Ornithodorus moubata* (Kovar et al., 2000). Infection with *R. rickettsii* also induced expression of PGRP CDSs in MG of both *A. sculptum* and *A. aureolatum*. Depending on the presence of an amidase catalytic site, those proteins are classified into non-catalytic or catalytic. Non-catalytic PGRPs function as pathogen pattern recognition receptors and activate the Toll and Imd pathways in *Drosophila* upon infection, while catalytic PGRPs cleaves peptidoglycan, acting, therefore, as negative regulators of the immune response (by removing peptidoglycan) or as effectors (by killing bacteria) (Palmer and Jiggins, 2015). The PGRPs of both *A. sculptum* and *A. aureolatum* exhibit amidase catalytic site, suggesting they might play a role as effectors or negative regulators of tick immune signaling pathways. One protein containing ML domain was also induced in *A. sculptum* by infection. Genes encoding ML-domain containing proteins were detected to be expressed in MG of *I. ricinus* (Rudenko et al., 2005; Horackova et al., 2010). The function of such proteins in tick gut is not known, but is likely that they might be involved in response against invader pathogens and/or lipid metabolism (Inohara and Nunez, 2002).

The production of AMPs in tick MG is important, as the slow intracellular digestion of nutrients and neutral pH may favor the proliferation of microorganisms (Kopacek et al., 2010; Hajdusek et al., 2013). One lysozyme CDS was induced by infection in both *A. sculptum* and *A. aureolatum*. In *A. sculptum*, infection also induced the expression of two CDSs that code AMPs similar to the defensin of *A. americanum*, named amercin (Todd et al., 2007). Conversely, sequences encoding one 5.3-kDa AMP and one AMP with chymotrypsin-elastase inhibitory activity, similar to the ixodidin of *R. microplus* (Fogaca et al., 2006), were downregulated in the MG of infected *A. aureolatum*. These results suggest that the MG of *A. sculptum* might be more hostile to *R. rickettsii* than the MG of *A. aureolatum*. Then, functional studies are required to determine the importance of AMPs in protection of *Amblyomma* ticks against rickettsiae.

In insects, it is well-known that transcription of AMPs, as well as other immune effectors, is mainly regulated by the intracellular signaling pathways Toll, Immune deficiency (Imd), JNK (Jun-N-terminal kinase), and Jak/Stat (Ferrandon et al., 2007; Souza-Neto et al., 2009; Kleino and Silverman, 2014). Much less information is available on the immune signaling pathways in ticks. Immune signaling pathway components were identified in the genome of *I. scapularis* (Smith and Pal, 2014; Kotsyfakis et al., 2015; Gulia-Nuss et al., 2016). It was recently reported that Toll and Jak-Stat insect immune signaling pathway components are well conserved in ticks. Conversely, ticks lack some IMD signaling pathway components, such as the adaptor protein IMD, its associated molecule FAAD (Fas associated protein with death domain), the caspase DREDD (death related ced-3/Nedd2-like), and the negative regulators Pirk (poor IMD response upon knock-in) and Dnr1 (defense repressor 1) (Rosa et al., 2016). This same study has also shown that *A. marginale* downregulates the expression of immune signaling pathway components in an embryonic cell line (BME26) of *R. microplus*, its biological vector, while other microbial stimuli, including *R. rickettsii*, induce their expression. In addition, it was previously reported that the Jak/Stat pathway regulates the expression of 5.3-kDa AMP, which is essential to prevent *A. phagocytophilum* infection in *I. scapularis* (Liu et al., 2012). Jak/Stat pathway was also enrolled in controlling the production of peritrofin-1, which is essential for the successful colonization of *I. scapularis* MG by *B. burgdorferi* (Narasimhan et al., 2014). Interestingly, TRAF, a downstream member of the Toll pathway, was differentially expressed in both *A. sculptum* and *A. aureolatum*. Therefore, functional studies are warranted to determine the role played by immune pathways in the control of rickettsial infection in *Amblyomma* ticks.

In conclusion, the current study shows that infection with *R. rickettsii* elicits a distinct transcriptional profile in the MG of *Amblyomma sculptum* and *A. aureolatum*, which might be responsible for their differences in susceptibility to infection. The proteins encoded by differentially expressed CDSs should be functionally characterized and might be targets for development of blocking vaccines. As the genome of *A. sculptum* and *A. aureolatum* is unavailable, this study provides a rich source of sequences in the genus *Amblyomma*.

## Author contributions

Designed the experiments: ACF. Generated biological samples: LM, MG, and FC. Performed the experiments: LM and MG. Analyzed RT-qPCR data: LM, MG, and AF. Performed bioinformatics data analysis: JR. Performed statistic data analysis: JR, AF. Contributed reagents/materials/analysis tools: JR, AF, ML, SD, and ACF. Wrote the paper: LM, MG, and ACF. All authors read and approved the final manuscript.

## Funding

This work was supported by funds from the São Paulo Research Foundation (FAPESP; Grants 2008/053570-0, 2013/26450-2, and 2014/11513-1), the National Council for Scientific and Technological Development [CNPq; grants CNPq 573959/2008-0; The National Institutes of Science and Technology Program in Molecular Entomology (INCT-EM)], the Coordination for the Improvement of Higher Education Personnel (CAPES), and the Provost for Research of the University of São Paulo [Research Support Center on Bioactive Molecules from Arthropod Vectors (NAP-MOBIARVE 12.1.17661.1.7)]. MG and LM were respectively supported by doctoral and master's fellowships from FAPESP.

### Conflict of interest statement

The authors declare that the research was conducted in the absence of any commercial or financial relationships that could be construed as a potential conflict of interest.

## Abbreviations

MG: Midgut
SG: salivary glands
AsC: *Amblyomma sculptum* control
AaC: *Amblyomma aureolatum* control
AsI: *Amblyomma sculptum* infected
AaI: *Amblyomma aureolatum* infected
CDSs: coding sequences.
