# Supplementary figures and images for: The Distinct Transcriptional Response of the Midgut of Amblyomma sculptum and Amblyomma aureolatum Ticks to Rickettsia rickettsii Correlates to Their Differences in Susceptibility to Infection
Source: Front Cell Infect Microbiol. 2017 Apr 28;7:129. doi: 10.3389/fcimb.2017.00129 (PMC5409265; doi:10.3389/fcimb.2017.00129)

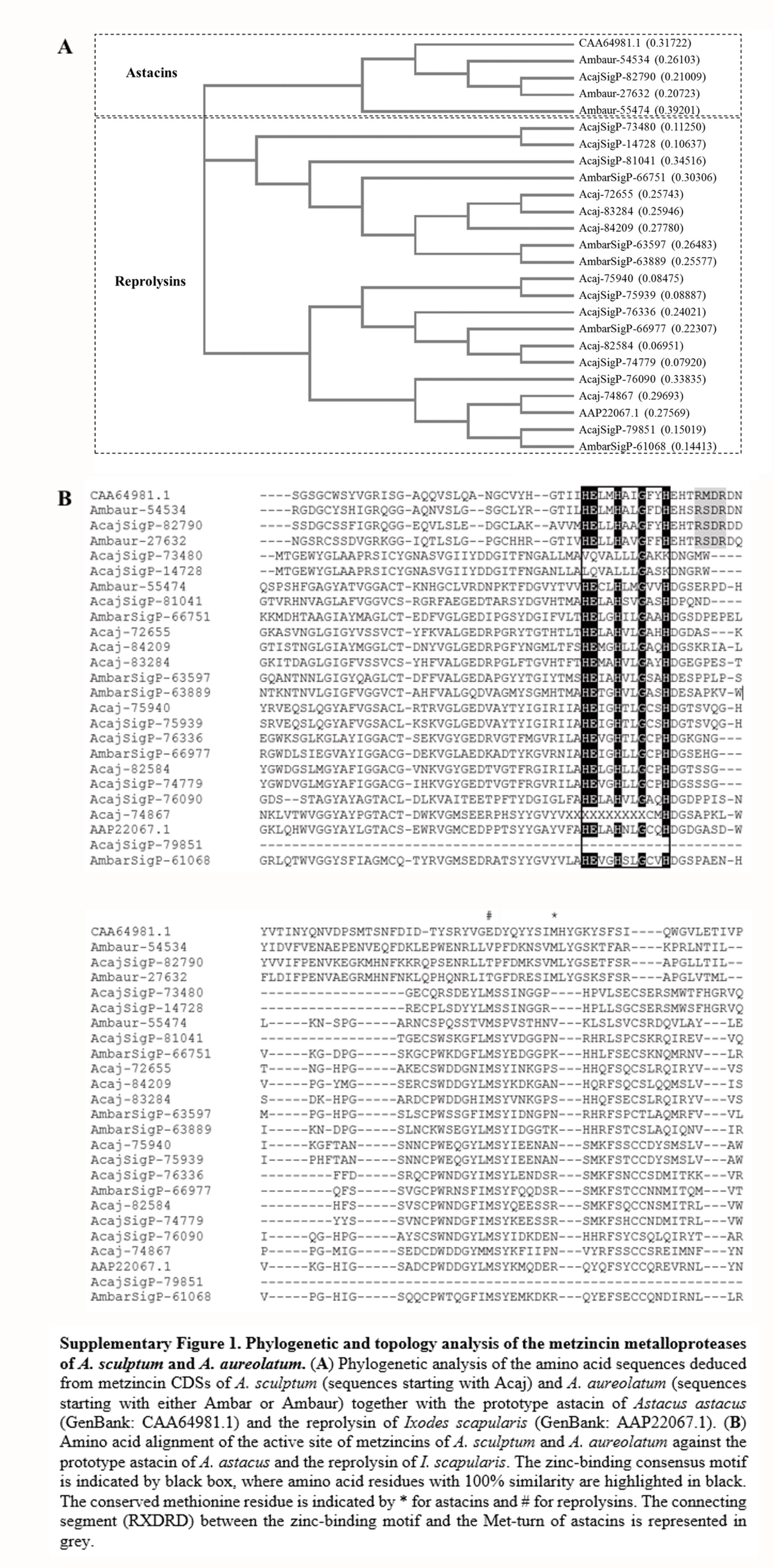

Supplement: Supplementary file 6 [file Image1.TIF]
